# Application of propidium monoazide quantitative PCR to discriminate of infectious African swine fever viruses

**DOI:** 10.3389/fmicb.2023.1290302

**Published:** 2024-01-10

**Authors:** Yang Li, Zewei Wang, Jie Qing, Dajun Hu, Hong Trang Vo, Kim Thanh Thi, Xinglong Wang, Xiaowen Li

**Affiliations:** ^1^Xiajin New Hope Liuhe Agriculture and Animal Husbandry Co., Ltd., (Shandong Engineering Laboratory of Pig and Poultry Healthy Breeding and Disease Diagnosis Technology), Dezhou, China; ^2^New Hope Binh Phuoc livestock Co., Ltd., Huyen Hon Quan, Vietnam; ^3^College of Veterinary Medicine, Northwest A&F University, Xianyang, Yangling, China

**Keywords:** African swine fever virus, propidium monoazide, quantitative PCR, viability, persistence

## Abstract

**Introduction:**

The detection of African swine fever virus (ASFV) is commonly performed using quantitative real-time PCR (qPCR), a widely used virological method known for its high sensitivity and specificity. However, qPCR has a limitation in distinguishing between infectious and inactivated virus, which can lead to an overestimation of viral targets.

**Methods:**

To provide insights into ASFV infectivity, we evaluated the suitability of PMAxx, an improved version of propidium monoazide (PMA), as a means to differentiate between infectious and non-infectious ASFV. Pre-treatment with 50 μM PMAxx for 15 min significantly reduced the qPCR signal of ASFV in the live vaccine. Additionally, thermal treatment at 85°C for 5 min effectively inactivated the live ASFV in the vaccine. Based on a standard curve, the sensitivity of the PMAxx-qPCR assay was estimated to be approximately 10 copies/μL. Furthermore, we observed a strong agreement between the results obtained from PMAxx-qPCR and pig challenge experiments. Moreover, we utilized the PMAxx-qPCR assay to investigate the persistence of ASFV, revealing a close relationship between viral persistence and factors such as temperature and type of piggery materials.

**Conclusion:**

The findings of this study suggest that pre-treating viruses with PMAxx prior to qPCR is a reliable method for distinguishing between infectious and non-infectious ASFV. Thus, integrating of PMAxx-qPCR into routine diagnostic protocols holds potential for improving the interpretation of positive ASFV results obtained through qPCR.

## Introduction

African swine fever (ASF) is a devastating disease that affects domestic pigs and wild boars. ASF outbreaks are currently occurring in Africa, Eastern Europe and Asia, causing significant economic losses globally (Costard et al., 2009; Norbert Mwiine et al., 2019). The African swine fever virus (ASFV), the pathogen responsible for ASF, is the sole member of the *Asfarviridae* family. It has a double-stranded DNA genome of approximately 180–190 kb and encodes over 150 open reading frames (ORFs) (Dixon et al., 2013). ASFV can survive in the environment for extended periods and can be transmitted through infected tick bites, direct contact with infected pigs, and contaminated materials (Gaudreault et al., 2020; Pereira De Oliveira et al., 2020). Due to the lack of commercial vaccines in the past few decades, preventing severe ASF outbreaks heavily relies on restricting animal movements and culling infected herds (Zhang et al., 2020; Borca et al., 2021). Recently, a promising recombinant vaccine candidate, ASFV-G-1I177L, has been developed by deleting the I177L gene from the genome of the highly virulent ASFV Georgia strain (Borca et al., 2020, 2021; Tran et al., 2021, 2022). This attenuated vaccine has been authorized as the first commercial gene-modified live vaccine in Vietnam and has shown no residual toxicity in long-term clinical studies (Borca et al., 2023). However, the vaccine can be only given to pigs aged between 8 and 10 weeks according to the directions. The residual infectious particles in the environment poses significant challenges to ASF risk management in the clearance of ASFV.

Quantitative real-time polymerase chain reaction (qPCR) is highly sensitive and specific for detecting the presence of viral genomes (Choi and Jiang, 2005; Hamza et al., 2011). However, qPCR cannot differentiate between infectious and inactivated viruses to directly indicate infectivity (Fittipaldi et al., 2010). Methods that can rapidly provide information about viral infectivity are of interest, given that only active viruses pose a public health threat (Knight et al., 2013). Various methods have been employed to detect infectious viruses, including cytopathic effect, fluorescent microscopy, flow cytometry, and detection of genome or envelope integrity (Zeng et al., 2022). Detection methods involving cell culture are considered the gold standard for quantifying certain viral infectivity. However, ASFV cultivation requires costly primary porcine alveolar macrophages and is constrained to biosafety level 3 laboratories (Blackmer et al., 2000; Rodríguez et al., 2009; Hamza et al., 2011). Additionally, the proposal to analyze the integrity of viral genomes through PCR amplification of long target regions may be related to viral infectivity (Li et al., 2002; Simonet and Gantzer, 2006). Nevertheless, viral inactivation can occur without damaging the viral genome, limiting the general applicability of long target region PCR as a surrogate marker for viral infectivity (Hamza et al., 2011).

**FIGURE 1:**
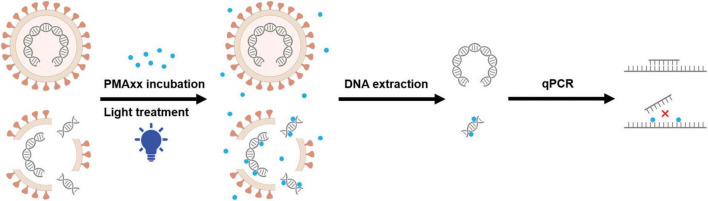
Proposed mechanism of PMAxx modification of dead viruses. The envelope impermeant PMAxx dye (purple dot) selectively penetrates dead viruses with compromised membranes. Upon exposure to light, PMAxx covalently modifies dissociated DNA. Genomic DNA was extracted from these samples, while some of the PMAxx-modified DNA became insoluble and was lost during DNA extraction. Subsequent qPCR amplification of modified DNA templates is inhibited, allowing selective quantification of DNA from viable viruses.

Cellular or envelope integrity is one of the characteristics used to distinguish between live and inactivated cells or enveloped viruses. One promising strategy to overcome the limitations of qPCR is pre-treating samples with photosensitizing dyes such as ethidium monoazide (EMA) and propidium monoazide (PMA) before qPCR (Parshionikar et al., 2010). This approach has been successfully used to differentiate infectious and non-infectious bacteria, protozoa, nematode eggs, fungi and viruses (Brescia et al., 2009; Fittipaldi et al., 2010; Graiver et al., 2010; Kim et al., 2011; Dreo et al., 2014). Theoretically, the dyes are membrane-impermeant and bind irreversibly to nucleic acids by photoactivation, leaving the DNA in viable cells intact (Nocker et al., 2006). Modified nucleic acid structures interfere with PCR amplification, resulting in reduced signal intensity in subsequent qPCR (Rudi et al., 2005; Nocker and Camper, 2009). Moreover, light exposure leads to the reaction of unbound excess dye with water molecules, preventing the purified DNA from being further modified in cells with intact cell membranes (Nocker and Camper, 2009). Some studies have shown significant DNA loss in the genomic DNA of live bacteria induced by EMA (Flekna et al., 2007), while PMA has been demonstrated to be more selective, only penetrating dead bacterial cells and not cells with intact membranes (Nocker et al., 2006). PMAxx, an improved version of PMA with a higher molecular charge, inhibits PCR amplification of modified DNA templates through a combination of removal of modified DNA during purification and inhibition of template amplification by DNA polymerases (Figure 1). In experimental bacterial strains, PMAxx increased the difference between live and dead bacteria by an additional 3 to 7 CT values compared to PMA (Nocker et al., 2006). Recently, Liu et al. conducted a study to investigate the addition of Triton X-100 for enhancing the penetration of PMAxx into inactivated ASFV virions, which may be helpful to interpret the results, though the infectivity of samples was still distinguishable by PMAxx-qPCR without the assistance of Triton X-100 (Liu et al., 2022). Moreover, these experiments were carried out solely under laboratory conditions and the validity of the results was not confirmed through animal inoculation.

The objective of this study was to evaluate the applicability of pure PMAxx pre-treatment for differentiating infectious and non-infectious ASFV and the correlation between PMAxx-qPCR detection results and animal challenges. We also used PMAxx-qPCR to assess the persistence of ASFV on commonly encountered materials in pig farms. Our data demonstrate that PMAxx-qPCR detection can improve the interpretation of ASFV qPCR-positive results and provide better risk management strategies.

## Materials and methods

### Preparation of ASFV samples

NAVET-ASFVAC vaccine containing live-attenuated ASFV-G-ΔI177L strain was purchased from Navetco National Veterinary Joint Stock Company (NAVETCO). The vaccine was diluted with saline at a twofold dilution ratio (ranging from 1:10 to 1:2560), aliquoted and stored at −80°C until use. The experiments of animal inoculation and viral persistence using NAVET-ASFVAC vaccine were performed in a fattening pig farm in Binh Phuoc province, Vietnam.

### PMAxx treatment

Each sample was divided into three portions: one portion was heat-inactivated at 85°C for 10 min, while the other two portions were kept at room temperature. PMAxx (Biotium, Inc., Hayward, CA, USA) was dissolved in deionized water to obtain a stock solution of 1 mM. Then, 10 μL of the PMAxx solution was added to 190 μL aliquots of both the non-heated and heat-treated samples, resulting in a final concentration of 50 μM. Additionally, 10 μL of deionized water was added to an untreated aliquot as a control template for standard qPCR. These three aliquots were incubated in the dark at 37°C for 15 min with occasional mixing to allow reagent penetration. Subsequently, the samples were irradiated with a PMA-Lite™ LED photoactivator (E90002, Biotium) at room temperature for 15 min. The experiment was repeated in triplicate, and DNA extraction of the samples was performed. Only when the CT values of the heat-treated-PMAxx treated aliquots were significantly higher than those of the PMAxx treated aliquots, the presence of ASFV viral particles was considered to be existed.

### Nucleic acid extraction

Samples were vortexed and centrifuged at 8,000 × *g* for 2 min. Genomic DNA from the samples (200 μL) was extracted using the Virus DNA Extraction Kit II (Geneaid, Taiwan) according to the instructions provided. The extracted nucleic acid was eluted in 50 μL elution buffer and stored at 4°C for subsequent qPCR analysis.

### Quantitative PCR (qPCR)

African swine fever virus-specific primers (forward: 5′-AAAATGATACGCAGCGAAC-3′, reverse: 5′-TTGTTTACCT GCTGTTTGGAT-3′) and a probe (5′-FAM-TTCACAGCATT TTCCCGAGAACT-BHQ1-3′) targeting the B646L gene were used for qPCR detection. A qPCR reaction mixture of 20 μL was prepared, containing 10 μL PerfectStart R II Probe qPCR SuperMix (TransGen Biotech, China), 0.5 μM primers and probe, 5 μL DNA template, and PCR-grade water. The reaction consisted of an initial denaturation step at 95°C for 10 min, followed by 40 cycles of denaturation at 95°C for 15 s, annealing at 60°C for 15 s, and extension at 72°C for 30 s. qPCR results were recorded using the Step One Plus™ Real-Time PCR System (ABI, 4376600).

### Standard curve

A standard plasmid containing the ASFV B646L gene was constructed as described previously (Li et al., 2022). Briefly, partial sequences of the B646L gene were amplified by PCR. The products and pMD18-T plasmids (D101A, Takara, Japan) were digested with the same restriction enzymes. The fragment was gel-purified and ligated to the vector using DNA ligase (C301-01, Vazyme) following standard procedures. Positive clones were screened and identified by sequencing. The standard curve was constructed using logarithmic 10-fold dilutions ranging from 2.5 × 10^7^ to 2.5 genome copies. The ASFV genome copy numbers corresponding to the copy numbers of the standard plasmid were calculated using the following formula:

$$C\,o\,p\,y\,n\,u\,m\,b\,e\,r=\frac{\text{m}\times 6.022\times 10^{23}}{1,840,105.22\times 1\times 10^{9}}$$

where m (/g) is the amount of plasmids measured using a BioSpec-nano Micro-volume UV-Vis Spectrophotometer (Shimadzu, Kyoto, Japan), 6.022 × 10^23^ is the Avogadro number, 1,840,105.22 (Da) is the molecular weight of standard plasmids calculated using the Sequence Manipulation Suite (Stothard, 2000), and 1 × 10^9^ is used to convert the molecular weight of the plasmids to nanograms. A fresh dilution set was prepared to construct the standard curve for each qPCR run, which could convert CT values obtained from qPCR analysis to ASFV genome equivalents.

### Animal inoculation

The NAVET-ASFVAC vaccine was diluted with saline (1:2560) and divided into four portions, with three portions (Groups B, C, and D) subjected to water bath heating at 85°C for 0.5, 1, and 10 min, respectively. The infectiousness of ASFV particles in the samples was quantified using PMA-qPCR. Then, each sample was injected into the muscles of three ASFV-negative fattening pigs. Throat swab samples were collected from each fattening pig 7 days later as previously described (Li et al., 2022) and ASFV DNA was measured using qPCR.

### Detection of ASFV persistence on the piggery materials

The vaccine was diluted with saline to a CT value of 25. Latex gloves and packaging bags from the pig farm were cut into squares with sides measuring 2 cm and soaked in the vaccine. Dry feed particles of approximately 1 cm in length were selected, and 50 μL of vaccine was added to each particle. The foam plastic heads of fertilization tubes were dipped into the vaccine. These materials were then thoroughly air-dried and placed in an incubator at temperatures of 4, 15, or 25°C for several days. The samples’ ASFV infectivity was measured by PMAxx-qPCR in three separate experiments after dissolution in 2 mL of saline. The experiments were performed in a fattening pig farm in Binh Phuoc province, Vietnam.

### Statistical analysis

The significance of differences between CT values was evaluated by unpaired Student’s *t*-test using GraphPad Prism v8.3.0. In all cases, a value of *P* < 0.05 was considered significant.

## Results

### Optimization of the experimental conditions

**FIGURE 2:**
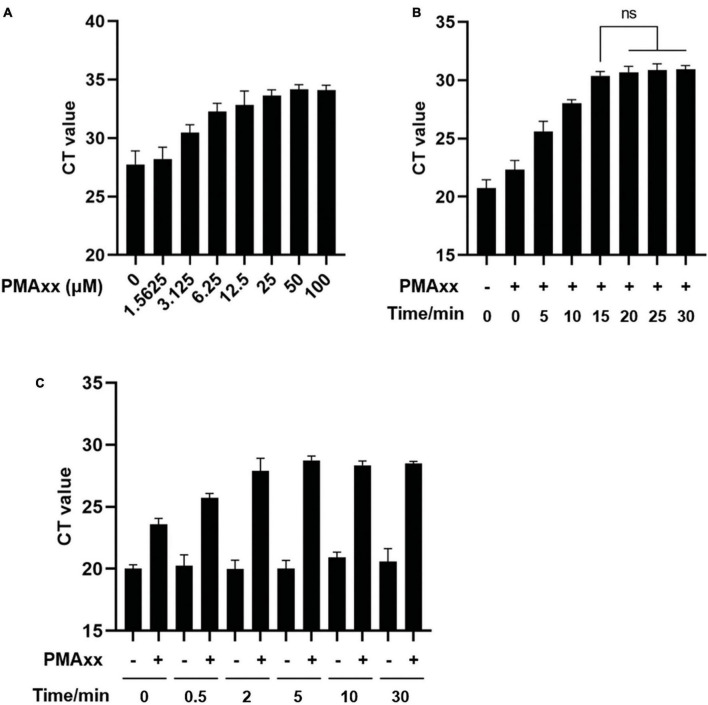
Optimization of PMAxx-qPCR assay conditions. **(A)** Vaccine samples containing ASFV-G-ΔI177L were treated with different concentrations of PMAxx-qPCR at 37°C for 15 min. Genomic DNA content was detected by qPCR using ASFV B646L gene-specific primers and probe. **(B)** Vaccine samples containing ASFV-G-ΔI177L were incubated with 50 μM PMAxx at 37°C for different times. ASFV DNA was detected by qPCR. **(C)** Vaccine samples containing ASFV-G-ΔI177L were heat-killed at 85°C for various times and then incubated with 50 μM PMAxx at 37°C for 15 min. ASFV DNA was detected by qPCR. All assays were performed in triplicate.

To determine the appropriate working concentration, various doses of PMAxx were added to ASFV-positive vaccine samples and exposed to light for 15 min. Subsequently, genomic DNA was extracted and subjected to qPCR using ASFV-specific primers and probes. The results displayed in Figure 2A demonstrated that increasing concentrations of PMAxx led to higher CT values, indicating greater inhibition of qPCR amplification. Concentrations ranging from 25 to 50 μM led to substantial inhibition, whereas higher concentrations did not entirely eliminate qPCR signals in samples with high ASFV content.

To optimize the efficacy of PMAxx-DNA cross-linking, ASFV nucleic acids were incubated with 50 μM PMAxx at 37°C for varying durations. As depicted in Figure 2B, inhibition increased with longer incubation times. Pre-treatment with PMAxx for 15 min yielded similar results as a 30-min pre-treatment, suggesting that a 15-min period achieved complete cross-linking of PMAxx with ASFV DNA.

For the preparation of an inactive ASFV control, genomic DNA solutions were subjected to thermal inactivation using water baths set at 85°C for different durations. Subsequently, PMAxx was added at a final concentration of 50 μM and incubated at 37°C for 15 min with periodic mixing. Figure 2C indicated that heat treatment of infectious ASFV for up to 30 min did not affect the qPCR CT values. However, the signal from PMAxx-treated DNA was reduced compared to untreated DNA at all-time points. Based on the degree of inhibition, a thermal inactivation period of 5–30 min efficiently inactivated ASFV and released DNA for subsequent reaction with PMAxx at 85°C.

To guarantee efficient cross-linking, a PMAxx concentration of 50 μM, an incubation time of 15 min, and the thermal inactivation at 85°C for 10 min were selected for subsequent experiments.

### Sensitivity of the PMAxx-qPCR assay

**FIGURE 3:**
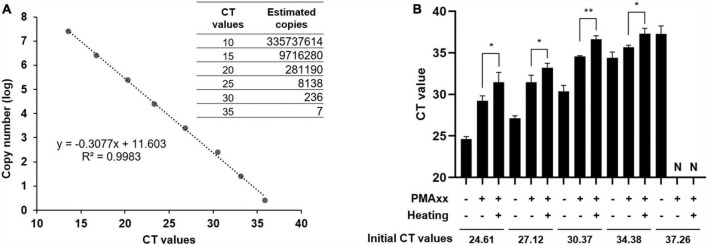
PMAxx-qPCR sensitivity estimated from the standard curve. **(A)** Correlation between ASFV copy numbers and CT values was established using ASFV B646L gene standard plasmids. **(B)** Vaccine samples containing ASFV-G-ΔI177L were subjected to PMAxx-qPCR assay. All assays were performed in triplicate. N, negative. *P < 0.05, **P < 0.01.

To assess the sensitivity of the PMAxx-qPCR assay, a standard curve was generated using a 10-fold serial dilution of standard plasmids containing ASFV B646L partial sequences. The CT values were plotted against the logarithm of the standard plasmid copies to perform linear regression analysis (Figure 3A). The experimental points aligned in a straight line with a high correlation coefficient (*R*^2^ = 0.9983), indicating accurate prediction. The limit of quantification for the PMAxx-qPCR assay was determined by testing serially diluted vaccine samples containing ASFV-G-ΔI177L. As shown in Figure 3B, samples with a mean CT value of 34.38 were identified as having infectious ASFV by PMAxx-qPCR, corresponding to approximately 10.57 copies of total ASFV according to the linear relationship in Figure 3A. Therefore, the detection limit of this assay was approximately 10 copies/μL when testing for infectious ASFV in this vaccine.

### Concordance between PMAxx-qPCR assay and pig challenge results

**FIGURE 4:**
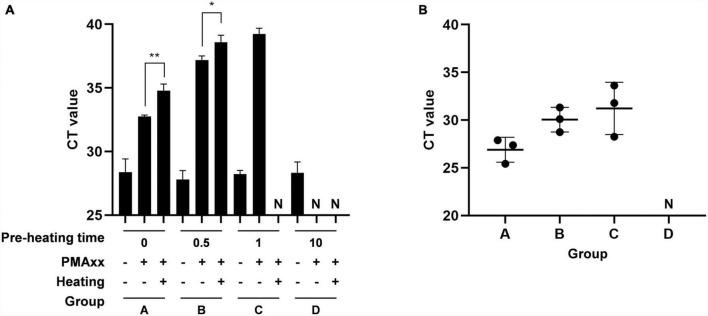
Comparison of agreement between PMAxx-qPCR and inoculation of animals. The vaccine containing ASFV-G-ΔI177L was heated at 85°C for different times. **(A)** The infectivity of samples was quantified by PMAxx-qPCR. **(B)** Each sample was intramuscularly injected into the neck of three ASFV-negative fatting pigs. Throat swab samples were collected from all pigs after 7 days and ASFV DNA levels were determined by qPCR. N, negative. *P < 0.05, **P < 0.01.

To validate the reliability of the PMAxx-qPCR assay, the diluted vaccine containing viable ASFV was subjected to different durations of heat treatment at 85°C. The PMAxx-qPCR assay was then performed to detect the ASFV activity in all samples. Figure 4A demonstrated that heat treatment for 0.5 min (B) and 1 min (C) did not completely inactivate ASFV, while samples heated for 10 min (D) showed no infectious ASFV. Each sample was intramuscularly injected into three ASFV-negative fattening pigs, and their throat swab samples was collected after 7 days. ASFV genomic DNA was quantified using specific primers and probes targeting the B646L gene. Figure 4B revealed that pigs challenged with sample A (positive control), B, or C were successfully infected with ASFV, while pigs injected with sample D remained ASFV-negative throughout the experiment. These results demonstrated a strong agreement between the PMAxx-qPCR assay and pig challenge results.

### Application of PMAxx-qPCR to determine ASFV survival time on piggery materials

African swine fever virus can be transmitted to pigs through close contact with contaminated piggery supplies (Gaudreault et al., 2020). In this study, the persistence of ASFV particles on the surface of piggery materials at different temperatures was estimated.

Different materials, including dry feed, the head of fertilization tubes, latex gloves, and packaging bags were soaked in ASFV vaccine, air-dried, and stored at different temperatures for several days. ASFV on the materials was eluted with saline, and viral infectivity was measured using PMAxx-qPCR (Table 1). Overall, the survival time of infectious ASFV on the four different matrices decreased with increasing temperature (from 20–45 days at 4°C to 7–15 days at 25°C). The fertilization tubes showed the greatest delay in ASFV survival time from 25 to 4°C (30 days), while latex gloves exhibited the shortest change of survival time (13 days) when the temperature decreased. Infectious ASFV persisted for 20 days on latex gloves at 4°C, whereas the survival time on fertilization tubes was 45 days at the same temperature. Significant differences in ASFV survival time were observed between latex gloves and fertilization tubes at 25°C and 15°C, but no significant difference was found between dry feeds and packaging bags at all temperatures. These findings indicate that the persistence of ASFV infectivity varied depending on the type of piggery material and temperature.

**TABLE 1:** Detection of ASFV survival time on the piggery materials by PMAxx-qPCR.

Temperature	Materials	1	2	3	5	7	10	15	20	25	30	35	40	45	50	55	60
25°C	Dry feed	+	+	+	+	+	+	−	−	−							
	Fertilization tubes	+	+	+	+	+	+	+	−	−	−						
	Latex gloves	+	+	+	+	+	−	−	−								
	Packaging bags	+	+	+	+	+	+	−	−	−							
15°C	Dry feed	+	+	+	+	+	+	+	−	−	−						
	Fertilization tubes	+	+	+	+	+	+	+	+	−	−	−					
	Latex gloves	+	+	+	+	+	+	+	−	−	−						
	Packaging bags	+	+	+	+	+	+	+	−	−	−						
4°C	Dry feed	+	+	+	+	+	+	+	+	+	+	−	−	−			
	Fertilization tubes	+	+	+	+	+	+	+	+	+	+	+	+	+	−	−	−
	Latex gloves	+	+	+	+	+	+	+	+	−	−	−					
	Packaging bags	+	+	+	+	+	+	+	+	+	+	−	−	−			

Piggery materials, including latex gloves, dry feed, packing bags and the head of fertilization tubes were soaked using vaccine of ASFV-G-ΔI177L. The materials were then laid out to dry thoroughly and stored at 25, 15, or 4°C for several days. ASFV infectivity was measured by PMAxx-qPCR until no infectious ASFV was detected in three continuous sampling. All experiments were performed in duplicate under same conditions and representative results are shown. +: infectious ASFV positive; −: infectious ASFV negative.

## Discussion

Quantitative PCR-based methods are commonly used for risk assessment in pig production. However, samples from farms often contain a mixture of infectious and non-infectious causative agents, limiting the significance of qPCR without information on residual infectivity. Furthermore, incomplete or excessive disinfection is a prevalent issue due to the lack of information on disinfection efficiency, resulting in residual live virus and failed reproduction, or wasted resources and environmental pollution. A technically easy, highly sensitive, widely applicable, and cost-effective method is required to determine virus infectivity. To address this, alternative methods have been proposed, such as using photoactivatable dyes to eliminate signal interference from inactivated viruses during PCR amplification. This technique has demonstrated success in detecting infectivity in many viruses (Parshionikar et al., 2010; Hamza et al., 2011; Sanchez et al., 2012; Leifels et al., 2015; Prevost et al., 2016; Randazzo et al., 2016).

One photoactivatable dye that has garnered significant attention, particularly for its potential use in assessing ASFV infectivity, is PMAxx. A recent review highlighted the promising application prospects of a rapid infectious ASFV detection technology based on PMA pre-treatment, which could greatly enhance various aspects of ASF prevention and control, including epidemic surveillance, disinfection treatment, and drug development (Zeng et al., 2022). In our study, we aimed to demonstrate the potential of PMAxx pre-treatment in distinguishing between infectious and non-infectious ASFV strains in vaccine. We successfully detected as few as 10 copies/μL of infectious ASFV using PMAxx-qPCR. Furthermore, there was a strong correlation between the results of PMAxx-qPCR and animal inoculation (Figure 4). Hence, the PMAxx-qPCR assay may serve as a rapid and cost-effective analytical tool for assessing the efficacy of virus-inactivating disinfectants by monitoring capsid damage. By employing PMAxx-qPCR, the appropriate working concentration of disinfectants could be determined, ensuring effective and economical usage. It should be noted we utilized a vaccine containing ASFV-G-ΔI177L as the sole material to develop the method. This choice was made in accordance with biosafety management requirements. However, for potential clinical application, it is crucial to assess the applicability of the developed method to clinical samples such as blood, saliva, and environmental samples in future studies. What’s more, PMA is not suitable for monitoring UV irradiation of bacteria and viruses. This is because viability dyes rely on membrane integrity as a viability criterion, whereas UV light primarily damages viral nucleic acids (Nocker et al., 2006; Leifels et al., 2015). Similarly, PMAxx-qPCR is not applicable to non-enveloped viruses.

In the case of thermally inactivated ASFV, theoretically, the addition of PMAxx should completely eliminate the qPCR signal. However, even with relatively high levels of PMAxx, CT values could still be detected in the presence of high viral genome concentrations (Figure 2). These findings align with observations in viability qPCR, where complete prevention of PCR amplification of thermally inactivated viruses is challenging (Leifels et al., 2015; Moreno et al., 2015; Randazzo et al., 2016). The incomplete inhibition could be attributed to several factors. Firstly, it may stem from the limited concentration of PMAxx, which aims to effectively eliminate non-infectious ASFV genomic DNA while avoiding potential DNA loss due to overconcentration. Moreover, the background levels could be influenced by the target sequence, amplicon size, incubation temperature, and secondary structure of the viral genome (Contreras et al., 2011; Soejima et al., 2011; Schnetzinger et al., 2013; Leifels et al., 2015; Prevost et al., 2016). Therefore, further studies should focus on optimizing the working conditions of PMAxx to maximize the differentiation between signals from infectious and inactivated viruses. This includes determining the optimal concentration, incubation time, and considering the addition of Triton, PMA enhancer, and protease K (Randazzo et al., 2016).

The estimated survival time of infectious ASFV is affected by several environmental factors, including pH, temperature, type of fomite, light exposure, and the presence of viral aggregates (Arzumanyan et al., 2021; Nuanualsuwan et al., 2022). Previous studies have evaluated the persistence of infectious ASFV on different materials under ambient temperatures. At 20°C, infectious ASFV was detected in complete feed until 1 dpi, in soybean meal until at least 21 dpi and in corncob particles until 1 dpi (Niederwerder et al., 2022). ASFV survived longer in pork (18–83 days) than in tissues (9–17 days) and plasma (14 days) at room temperature (Petrini et al., 2019; Mazur-Panasiuk and Wozniakowski, 2020; Fischer et al., 2021). Additionally, porous materials, such as rubber and cellulose paper, supported ASFV viability for longer periods (14–22 days) than non-porous materials, like glass and metal (11–17 days) at 25°C (Nuanualsuwan et al., 2022). Infectious ASFV has also been detected in sterile sand for at least 3 weeks, beach sand for up to 2 weeks, yard soil for 1 week, and swamp soil for 3 days (Carlson et al., 2020). However, farmers are particularly concerned about the survival time of live ASFV on daily input materials accessible to pigs. In our study, we estimated that ASFV remained infectious on the surface of piggery materials for 7–15 days at 25°C (Table 1), which aligns with previous research on other materials. Furthermore, we observed variations in the persistence of ASFV on four matrices. The causes of these differences remain unclear, but it is likely linked to differences in their physicochemical properties, such as the micropore size in the head of fertilization tubes and the watertightness of latex gloves. Nonetheless, it is essential to disinfect piggery materials thoroughly as they are significant carriers of ASFV between domestic pigs. For those materials that cannot be conventionally disinfected, sufficient static storage before transport to pig farms is a viable option. The duration of static storage should increase as the ambient temperature decreases.

## Conclusion

This study aimed to develop and optimize the PMAxx-qPCR assay for effective discrimination of infectious ASFV. The authenticity of the PMAxx-qPCR assay was verified by animal inoculation experiments. Furthermore, the persistence of ASFV was assessed on various surfaces, including latex gloves, dry feed, packaging bags, and fertilization tubes. The results revealed that the longevity of infectious ASFV decreased with rising temperatures on the four different matrices tested. Notably, the porous head of the fertilization tube exhibited higher stability than the waterproof latex gloves. Based on these findings, it can be concluded that the PMAxx-qPCR assay holds promise as an alternative method for assessing ASFV infectivity. Its integration into routine diagnostics can significantly enhance the interpretation of positive ASFV results, leading to improved accuracy in identifying infected cases.

## Data availability statement

The original contributions presented in this study are included in the article/supplementary material, further inquiries can be directed to the corresponding authors.

## Ethics statement

The animal study was approved by the Ethics Committee at Northwest A&F University. The study was conducted in accordance with the local legislation and institutional requirements.

## Author contributions

YL: Writing – original draft, Project administration, Methodology, Visualization. ZW: Methodology, Visualization, Writing – review and editing. JQ: Methodology, Visualization, Writing – review and editing. DH: Resources, Supervision, Validation, Writing – review and editing. HV: Data curation, Writing – review and editing. KT: Data curation, Writing – review and editing. XW: Supervision, Writing – review and editing. XL: Conceptualization, Funding acquisition, Writing – review and editing.
